# Tempol, a Membrane-Permeable Radical Scavenger, Exhibits Anti-Inflammatory and Cardioprotective Effects in the Cerulein-Induced Pancreatitis Rat Model

**DOI:** 10.1155/2016/4139851

**Published:** 2015-12-07

**Authors:** Andrzej Marciniak, Beata Walczyna, Grażyna Rajtar, Sebastian Marciniak, Andrzej Wojtak, Katarzyna Lasiecka

**Affiliations:** ^1^Chair and Department of Pathophysiology, Medical University of Lublin, 20-090 Lublin, Poland; ^2^Chair and Department of Clinical Pathomorphology, Medical University of Lublin, 20-090 Lublin, Poland; ^3^Department of Pharmacology, Medical University of Lublin, 20-093 Lublin, Poland; ^4^Chair and Department of Vascular Surgery and Angiology, Medical University of Lublin, 20-081 Lublin, Poland; ^5^Department of Pharmacology, University of Life Sciences in Lublin, 20-033 Lublin, Poland

## Abstract

To date, it remains unclear whether mild form of acute pancreatitis (AP) may cause myocardial damage which may be asymptomatic for a long time. Pathogenesis of AP-related cardiac injury may be attributed in part to ROS/RNS overproduction. The aim of the present study was to evaluate the oxidative stress changes in both the pancreas and the heart and to estimate the protective effects of 1-oxyl-2,2,6,6-tetramethyl-4-hydroxypiperidine (tempol) at the early phase of AP. Cerulein-induced AP led to the development of acute edematous pancreatitis with a significant decrease in the level of sulfhydryl (–SH) groups (oxidation marker) both in heart and in pancreatic tissues as well as a substantial increase in plasma creatine kinase isoenzyme (CK-MB) activity (marker of the heart muscle lesion) which confirmed the role of oxidative stress in the pathogenesis of cardiac damage. The tempol treatment significantly reduced the intensity of inflammation and oxidative damage and decreased the morphological evidence of pancreas injury at early AP stages. Moreover, it markedly attenuated AP-induced cardiac damage revealed by normalization of the –SH group levels and CK-MB activity. On the basis of these studies, it is possible to conclude that tempol has a profound protective effect against cardiac and pancreatic damage induced by AP.

## 1. Introduction

Despite great advancement in therapeutics, treatment for acute pancreatitis (AP) has not significantly changed for many years and the illness characterizes itself with a substantial percentage of complications and mortality with its pathogenesis is not fully recognized yet. Acute pancreatitis (AP) is an illness which may lead to general inflammatory reaction, which in turn may result in the development of multiple organ dysfunction syndrome [1, 2]. The pathogenesis of systemic complications of AP is still under intensive investigation. The clinical and experimental researches conducted so far have revealed the complex pathogenesis of AP and a serious role of oxidative and antioxidative processes in the development of this fatal illness. The uncontrolled generation of reactive oxygen and nitrogen species (ROS/RNS) during AP may lead to oxidative damage both in the pancreas and distant organs [3–5]. ROS/RNS are implicated in the pathophysiology of diverse cardiovascular diseases. These also appear to be common mediators of apoptosis, directly or via the formation of lipid peroxidation and lipid hydroperoxides [6–8].

To date, it remains unclear whether mild form of AP may cause myocardial damage which may remain asymptomatic for a long time. Hypothetical mechanisms have been proposed to explain pathogenesis of AP-related cardiac injury which may be attributed in part to ROS/RNS and inflammatory cytokines overproduction [9–12]. Thus, cytoprotective/antioxidant treatment may prevent development of cardiac injury at the early phase of AP.

4-Hydroxy-2,2,6,6-tetramethylpiperidine-1-oxyl (tempol) is a membrane-permeable aminoxyl-type free radical scavenger, with unique antioxidant properties. This compound exhibits a superoxide dismutase mimetic action to degrade superoxide radical, thereby preventing the formation of peroxynitrite. The advantageous effect of aminoxyl action is also caused by inhibiting Fenton's reaction catalyzed by Fe^2+^ ions [13]. Preclinical studies, using a variety of rat models, have shown the potential for many clinical applications, including control of hypertension and weight gain, protection against ionizing radiation and DNA damage, and prevention of doxorubicin treatment-related cardiomyopathy [13–16].

Therefore the aim of the present study was to evaluate the oxidative stress in the pancreas and the heart and to estimate the protective effects of tempol at the early phase of cerulein-induced AP.

## 2. Materials and Methods

### 2.1. Reagents

The reagents, hydrochloric acid, ferric chloride, sodium acetate, glacial acetic acid, and ferrous sulphate, were of analytical or HPLC grade. 2,4,6-Tris(2-pyridyl)-*s*-triazine (TPTZ, 99%), cerulein, and 4-hydroxy-TEMPO were purchased from Sigma-Aldrich, Co. (St. Louis, MO, USA). Hydrochloric acid, sodium acetate, sodium carbonate, iron(III) chloride hexahydrate (FeCl_3_·6H_2_O), iron(II) sulfate heptahydrate (FeSO_4_·7H_2_O), and methanol (99.8%) were obtained from POCH (Gliwice, Poland). Redistilled water was used for the preparation of solutions.

### 2.2. Animals and Experimental Model

The study was carried out on 24 male Wistar rats, 8–10 weeks old, weighing 180–200 g and obtained from a licensed breeder. The study was approved by the Ethical Committee for Animal Experimentation of the Medical University of Lublin (Licence number: 136). The rats were kept on standard rat chow and fasted overnight before the experiment with water* ad libitum*.

Acute pancreatitis was induced as previously described [17–19] with minor modifications. Four subcutaneous injections of cerulein (15 *μ*g kg^−1^ body weight) or vehicles (normal saline) were given consecutively at hour intervals.

Rats were randomly divided into four groups (*n* = 6 per group): Group I (control); Group II (tempol) treated with tempol (given in the 1st, 2nd, and 3rd hours after the first saline injection); Group III (cerulein), cerulein-induced pancreatitis without treatment; and Group IV (tempol + cerulein), cerulein-induced pancreatitis treated with tempol (3 × 100 mg/kg, given in the 1st, 2nd, and 3rd hours after the first cerulein injection).

We used three multiple injections of tempol to provide a maximal protective effect against cerulein-induced pancreatitis. The dose of tempol was chosen on the basis of a previous animal studies that showed that similar high doses were well tolerated [13, 20–22].

After 6 hours the rats were anaesthetized with thiopental sodium (50 mg/kg), the abdominal cavity was opened, and blood was collected from the abdominal aorta into tubes containing EDTA or serum-separating gel. The blood was centrifuged at 3000 ×g for 10 min. Plasma and serum were frozen and kept at −80°C until assayed. After blood withdrawal, the pancreas and heart were carefully dissected out, rinsed with saline, blotted on paper, weighed, and divided into samples. For biochemical analyses, tissue blocks of both pancreas and heart were snap-frozen in liquid nitrogen and stored at −80°C before homogenate preparation.

The specific parameters that were evaluated to assess the degree of pancreatic inflammation included serum amylase levels, pancreatic water content, and histological characteristics of pancreatic tissue (by fixation and staining with hematoxylin and eosin (H&E), followed by examination by light microscopy). Plasma creatine kinase isoenzyme (CK-MB) and markers for oxidative damage, ferric reducing ability of plasma (FRAP) and malondialdehyde and 4-hydroxynonenal (MDA + 4-HNE) and total sulfhydryl (–SH) groups, in pancreas and heart were determined.

### 2.3. Measurement of Parameters for Evaluating AP

#### 2.3.1. Determination of Pancreatic Water Content

The water content of the pancreas was used as an index of pancreatic edema. It was quantified by weighing the freshly harvested tissue (wet weight) and compared with the weight of the same sample after desiccation at 160°C for 48 h (dry weight) [23]. The results were calculated and expressed as a percentage ((wet weight − dry weight) × 100/wet weight).

#### 2.3.2. Serum Amylase

Serum amylase was measured by standard photometric reaction with a commercially available kit (Alpha Diagnostics), using a Shimadzu spectrophotometer (Shimadzu Corporation, Japan) according to the manufacturer's instructions.

#### 2.3.3. Histology

The pancreas was immediately rinsed and fixed in 10% buffered formalin solution. Pancreatic scrapes were embedded in paraffin, cut and stained with hematoxylin and eosin (H&E), and graded in a blinded manner. For quantification of edema, vacuolization, inflammation (neutrophil infiltration), and necrosis, a modification of the score originally described by Tani et al. [24] was used, ranging from 0 to 3 (edema, vacuolization, inflammation, and necrosis).

### 2.4. Indices of Oxidative Stress

#### 2.4.1. The Antioxidant Capacity of Plasma

The antioxidant capacity of plasma samples was determined by the ferric-reducing antioxidant power (FRAP) assay of Benzie and Strain [25]. This method measures the ability of the antioxidants contained in a sample to reduce ferric to ferrous ion which at low pH forms a coloured ferrous-tripyridyltriazine complex that absorbs light at 593 nm.

#### 2.4.2. Determination of Lipid Peroxidation

The levels of malondialdehyde (MDA) and 4-hydroxynonenal (4-HNE) in the tissues of the pancreas and the heart were determined to assess lipid peroxidation. In brief, samples of tissues weighing about 300 mg were homogenized in the phosphate buffer (20 mM, pH 7.4). Prior to homogenization, 10 *μ*L of 0.5 mol/L butylated hydroxytoluene in acetonitrile was added to every 1 mL of tissue homogenate for each sample to prevent tissue oxidation. Tissue samples were centrifuged (3000 ×g at 4°C for 10 min) and the supernatant was frozen at −80 until assay. MDA + 4-HNE were measured spectrophotometrically using a commercial kit Bioxytech LPO-586 (Oxis, Portland, USA) according to the manufacturer's protocol. This assay is based on the reaction of a chromogenic reagent N-methyl-2-phenylindole with MDA and 4-HNE at 45°C. This reaction yields a stable chromophore with maximal absorbance at 586 nm [26].

#### 2.4.3. Total Sulfhydryl (–SH) Groups Assay

The total –SH group content in homogenates was measured using DTNB (2, 2′-dinitro-5,5′-dithiodibenzoic acid) as described by Ellman [27]. DTNB can react with the thiol group to produce a colored compound that absorbs light at 405 nm. The rate of chromophore production is proportional to the concentration of total –SH within the sample. The values were expressed as nmol/mg protein.

### 2.5. Creatine Kinase Isoenzyme (CK-MB) Activity

Plasma CK-MB activity was measured by an automatic chemistry analyser using a Vitros CK slides (Ortho-Clinical Diagnostics, Inc., Rochester, NY) to monitor possible cardiotoxicity effect of AP.

### 2.6. Determination of Protein Concentration

Sample protein contents were estimated by Bradford's method using bovine serum albumin as a standard [28].

### 2.7. Statistical Analysis

Results are expressed as means ± SEM where appropriate. The histopathological data are expressed as median (with low and high ranges). Statistical analysis was performed using analysis of variance (ANOVA) followed by Kruskal-Wallis or Tukey's multiple range test as post hoc. Probability values less than 0.05 were considered significant. Data handling and statistics were performed using the statistical software package STATISTICA, version 10 (StatSoft Inc.).

## 3. Results

### 3.1. Effects of Tempol on Serum Amylase Level, Pancreatic Water Content, and Histopathological Changes in the Pancreas

**Figure 1:**
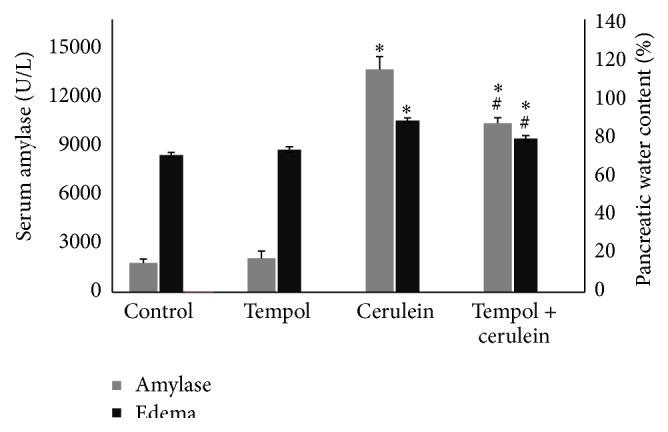
Effect of cerulein (15 *μ*g/kg/h for 4 h) given alone or in combination with tempol on serum amylase activity and pancreatic water content. Mean ± SEM. ^*∗*^ *p* < 0.05 compared with control. ^#^ *p* < 0.05 compared with cerulein given alone.

To study the role of tempol in cerulein-induced AP, blood amylase levels and pancreatic water content were initially determined as an indicator of severity of AP. Repeated injections of cerulein produced mild model of AP with the expected elevation in serum amylase and pancreatic water content 6 hrs later, both of which are highly significant when compared to those obtained in control groups (control; tempol). Administration of tempol (tempol + cerulein) reduced both cerulein-provoked blood amylase levels and pancreatic water content from 13882 ± 801 down to 10533 ± 343 U/L and from 88.1 ± 1.3% down to 78.9 ± 1.5%, respectively (Figure 1).

**Figure 2:**
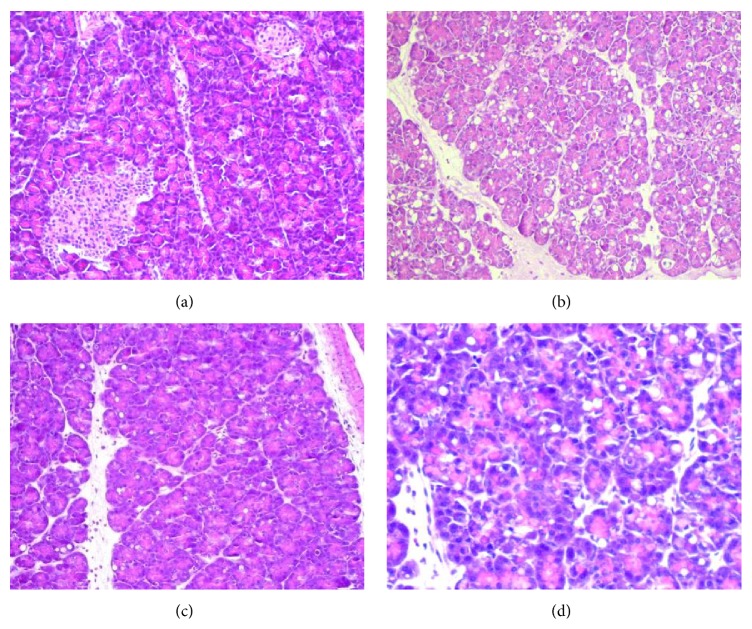
Histologic section of pancreas: (a) control rats—normal pancreatic structures were observed, (b) the rats subjected to cerulein-induced pancreatitis (AP) alone—evidences of edema, vacuolization, inflammatory cell infiltrate, and necrosis were noted, (c, d) the AP animals treated with tempol—note the significant reduction in edema, vacuolization, and inflammation, without any evidence of necrosis as compared to untreated animals (b). H&E staining; original magnification, ×20.

Morphological signs of AP were associated with biochemical markers of the severity of acute pancreatitis. Normal pancreatic microarchitecture was found in control groups (control; tempol; Table 1, Figure 2), whereas cerulein challenge caused destruction of the pancreatic tissue structure, characterized by vacuolization of acinar cells, perivascular infiltration of neutrophils, necrosis, and pancreatic edema formation. Treatment with tempol significantly reduced both the extent and the severity of the histological signs of pancreas injury (*p* < 0.05, Table 1 with exemplifying slides shown in Figure 2).

### 3.2. Oxidative Stress Evaluation

#### 3.2.1. Oxidative Injury Indices

**Figure 3:**
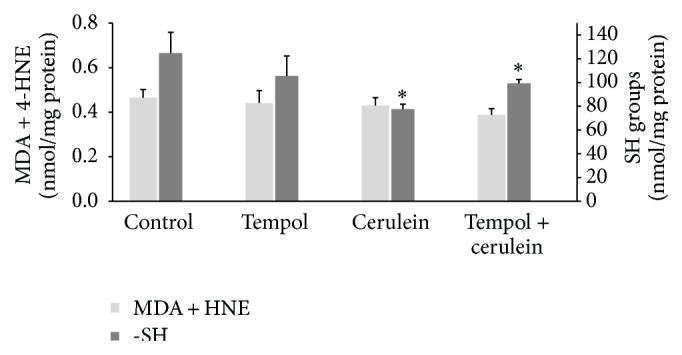
Effect of administration of tempol on oxidative stress parameters in pancreatic homogenates of rats with cerulein-induced pancreatitis. Mean ± SEM. ^*∗*^ *p* < 0.05 compared with control.

**Figure 4:**
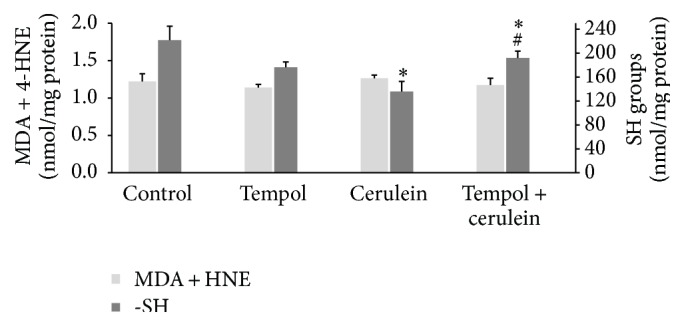
Effect of administration of tempol on oxidative stress parameters in heart homogenates of rats with cerulein-induced pancreatitis. Mean ± SEM. ^*∗*^ *p* < 0.05 compared with control. ^#^ *p* < 0.05 compared with cerulein given alone.

Cerulein challenge resulted in a significant decrease in the –SH group content in pancreas and heart in comparison to the control group values (77.5 ± 4.2 nmol/mg versus 124.7 ± 17.6 nmol/mg and 135.7 ± 17.0 nmol/mg versus 221.6 ± 23.5 nmol/mg, resp., *p* < 0.05). Treatment of cerulein-challenged animals with tempol partially restored the –SH group content in the pancreas (99.3 ± 3.2 nmol/mg versus 77.5 ± 4.2 nmol/mg) and also significantly enhanced –SH group content in the heart as compared to the cerulein-challenged rats (191.1 ± 11.4 nmol/mg versus 135.7 ± 17.0 nmol/mg, *p* < 0.05) (Figures 3 and 4). These findings suggest that tempol could have contributed to limiting oxidative damage caused by cerulein-induced AP. However, MDA + 4-HNE in heart and pancreatic tissues showed no difference among all four groups (*p* > 0.05; Figures 3 and 4).

#### 3.2.2. Total Antioxidant Capacity

**Figure 5:**
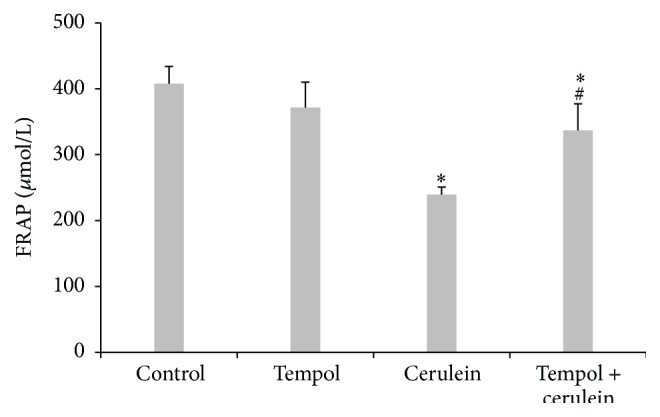
Effect of administration of tempol on plasma total antioxidant capacities measured by FRAP of rats with cerulein-induced pancreatitis. Mean ± SEM. ^*∗*^ *p* < 0.05 compared with control. ^#^ *p* < 0.05 compared with cerulein given alone.

Cerulein challenge also resulted in a significant reduction in plasma FRAP value as compared with control animals (240 ± 11 *μ*M versus 408 ± 26 *μ*M, *p* < 0.01): on the contrary tempol treatment reverted significantly this reduction in FRAP value caused by cerulein-induced AP (337 ± 40, *p* < 0.05) (Figure 5).

### 3.3. The Serum Levels of CK-MB

**Figure 6:**
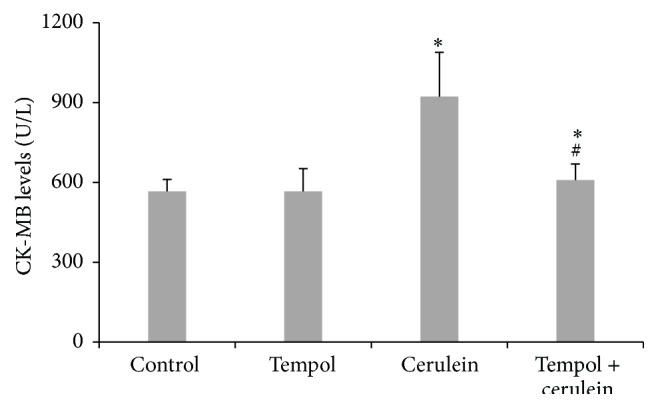
Effect of administration of tempol on activity of serum creatine kinase MB (CK-MB) of rats with cerulein-induced pancreatitis. Mean ± SEM. ^*∗*^ *p* < 0.05 compared with control. ^#^ *p* < 0.05 compared with cerulein given alone.

Tempol treatment alone did not induce any increase (*p* > 0.05) in CK-MB in comparison to the control group values. Cerulein challenge induced a significant increase in serum levels of CK-MB (a highly reliable biomarker of cardiac injury) compared with the control groups (922 ± 167 U/L versus 566 ± 45 U/L, *p* < 0.01). Treatment of cerulein-challenged animals with tempol (tempol + cerulein) markedly reduced CK-MB levels as compared to cerulein group (609 ± 60 U/L versus 922 ± 167 U/L, *p* < 0.01) (Figure 6).

## 4. Discussion

To date, it remains unclear whether mild oedematous form of AP may cause myocardial damage which may be asymptomatic for a long time. It has been also reported that cardiac disturbances such as electrocardiographic abnormalities may be associated with AP. Pathomechanisms of these processes have not been fully explained yet. Probably one of the major reasons for cardiac injury is the action of free radicals freed during the inflammatory processes. This leads to direct oxidative lesion of DNA and proteins and increased lipid peroxidation [29–31]. The other potential pathogenetic factors of cardiac disturbances include electrolyte alterations, systemic inflammatory response, or circulating proteolytic enzymes [32–35].

To study the involvement of oxidative stress in pancreatic and cardiac damage in the early stage of AP, we used a rat model of cerulein-induced AP. Hyperstimulation with this* CCK ortholog cerulein* leads to rapid development of mild, edematous form of experimental AP. The suggested mechanism of cerulein action involves production of ROS, activation of oxidant-sensitive transcription nuclear factor-*κ*B (NF-*κ*B), and release of proinflammatory cytokines [36, 37].

In the present study, we have observed that pancreatic overstimulation with cerulein causes a great intensification of vacuolization, leukocyte infiltration, and pancreatic edema evaluated on the basis of microscopic findings. The serum amylase and water content were also markedly elevated. The important finding of the present study is the observation that cerulein-induced AP led to a significant decrease in the level of sulfhydryl (–SH) group (representing the major pool of low molecular weight antioxidants) in both heart and pancreatic tissues as well as a marked increase of the level of CK-MB (marker of myocardial injury). We have also noted in cerulein group the decrease in FRAP value, which represents the combined potential of various antioxidants in the body. On the other hand, MDA + 4-HNE levels in both heart and pancreatic tissues are not significantly increased after 6 hours of experiment. This effect seems to be related to early onset of oxidative stress in the course of AP. Previous studies in the cerulein model found increased oxidative stress to occur very early, within the first 1-2 hours [38, 39]. It is possible that oxidative stress markers (MDA + 4-HNE) may be increased earlier and precede glutathione and low molecular antioxidants' decreases. Our results are in accordance with Kruse et al.'s findings [40]. They indicated no increase in markers of oxidative damage to DNA or oxidation of lipids measured by 7-hydro-8-oxo-29-deoxyguanosine and MDA + 4-HNE in cerulein model of acute pancreatitis, respectively.

Our study also confirms and extends previous reports showing that free radical scavenger tempol has an overall protective effect against pancreatic injury in a rat model of AP [41, 42]. The beneficial effect of administration of tempol was manifested by reduction in pancreatic edema, histological signs of pancreatic damage, and lower serum amylase level. This result is in accordance with the earlier reports that tempol attenuated the severity of AP. The experiments conducted by Śledziński et al. [42] pointed to a cytoprotective action of the nitrogen dioxide scavenger in the oxidative stress in the pancreas, which was induced with butyl hydroxide.

We have also noted that tempol treatment prevents the reduction of the radical scavenging activity in AP as measured by FRAP and –SH group content in both pancreas and heart. Moreover, treatment with tempol significantly attenuated increase in CK-MB activity caused by AP. However, it should be emphasized that tempol has other molecular mechanisms that possibly contribute to the protective effect against cerulein-induced AP injury. In other studies, Cuzzocrea et al. [43] showed that tempol inhibits the formation of peroxynitrous acid anion, which, in turn, prevents activation of poly(ADP ribose) synthetase (PARS) and lesions of the tissues connected with it. Additionally, by acting protectively on vascular endothelium, it inhibits the inflow of leukocytes to the inflammatory site [44]. We have observed a similar effect comparing the histological changes in pancreas in the cerulein groups of the animals. The degree of leukocyte infiltration of the pancreas in animals with AP that were given tempol was reduced as compared to the group of untreated animals with AP. In numerous studies using experimental models of AP, activated polymorphonuclear leukocytes (PMN) were implicated as the major source of oxidants in pancreatic and lung tissues [45–47]. Reduced leukocyte infiltration in the pancreas and limited activation of inflammatory cells may inhibit inflammation and oxidative stress, leading at the same time to the lowering of the degree of tissue damage [48].

## 5. Conclusions

Taken together, our study shows that tempol ameliorates significantly cerulein-induced AP, which has been confirmed by reduction of pancreatic edema, improvement in pancreatic histology, and reduction in serum amylase levels. Treatment with tempol had also a profound protective effect against cardiac damage induced by AP. These beneficial effects of this compound may be attributable in part to suppression of oxidative stress by alleviating protein oxidation through free radical scavenging at the early phase of AP. Therefore, antioxidant compounds may offer therapeutic approaches for AP and prevention of its cardiac complication. These data highlight the need for further research to identify the possible complication of mild AP and the potential benefits of early cytoprotective-specific therapies in this disease.

## Figures and Tables

**Table 1:** Histological findings in the pancreas observed in rats with or without cerulein and treated with saline or tempol.

	Edema (0–3)	Vacuolisation (0–3)	Infiltration (0–3)	Necrosis (0–3)
Control	0 (0-0)	0 (0-0)	0 (0-0)	0 (0-0)
Tempol	0 (0-0)	0 (0-0)	0 (0-0)	0 (0-0)
Cerulein	2.5 (2-3)^*∗*^	2.5 (1–3)^*∗*^	2 (1–3)^*∗*^	1 (0–2)^*∗*^
Tempol + cerulein	2 (1-2)^#**∗**^	1 (1-2)^**∗**#^	1 (0–2)^**∗**#^	0 (0-1)^**∗**#^

Median scores with ranges (min–max) of the results on 6 animals in each group are shown. ^*∗*^ *p* < 0.05 versus control, ^#^ *p* < 0.05 versus cerulein alone (analysis of variance followed by the Kruskal-Wallis test).
